# Beneficial effects and possible mechanism of intake coffee for COVID-19: A meta-analysis and molecular docking

**DOI:** 10.1097/MD.0000000000041550

**Published:** 2025-02-14

**Authors:** Yong-Zheng Fan, Yun-Li Duan, An-Na Zhang, Yu Wang

**Affiliations:** a Pharmacy Department, The 991st Hospital of Joint Logistic Support Force of People’s Liberation Army, Xiangyang, Hubei, China; b Teaching Department, Xiangyang No. 4 Middle School Compulsory Education Department, Xiangyang, Hubei, China.

**Keywords:** chlorogenic acid, coffee, COVID-19, meta, molecular docking

## Abstract

**Background:**

To systematically evaluate the effectiveness of regular coffee intake in the prevention or treatment of COVID-19 infection, and to explore its possible mechanism of action using computer molecular docking technology.

**Methods:**

We searched for relevant ClinicalTrials.gov, Cochrane Library, PubMed, Web of Science, Embase, and China Biomedicine, Wanfang, CNKI, VIP databases to summarize studies on the effectiveness of coffee in preventing or treating COVID-19. The search period lasted until August 1, 2024. The 2 researchers screened the literature and data using Rev Man 5.4 software (the Cochrane Collaboration, 2020) for data analysis and used Schrodinger 2018-1 software to explore possible mechanisms of action.

**Results:**

A total 5 studies with 39,290 participants were included. The results showed that compared with the control group that drank less or no coffee, the experimental group that drank more than 1 cup of coffee per day had significantly higher benefit rates (RD = 0.17, 95% confidence intervals [CI] = 0.08–0.27, *P* = .0005), including lower infection rates and improved recovery rates from COVID-19 (RD = 0.24, 95% CI = 0.13–0.35), *P* < .0001). Molecular docking showed that CGA and caffeine present in coffee could combine with key amino acid residues of ACE2 or 3CL proteins to form hydrogen bonds.

**Conclusions:**

Regular consumption of coffee may have certain preventive or therapeutic effects on COVID-19, and the mechanism of action may be that CGA or/caffeine in coffee may be related to the formation of hydrogen bonds by key amino acid residues such as ARG273/HIE345 of ACE2 and CYS145 of 3CL. Owing to the limited number and quality of the included studies, the effect evaluation needs to be further confirmed using clinical randomized controlled trials. The exact mechanism of action requires further verification at the molecular level, both inside and outside cells.

## 1. Introduction

The rapid spread of corona virus disease 2019 (COVID-19) called SARS-CoV-2, a member of the β-coronavirus family, is a single-stranded positive-sense RNA virus, approximately 30kb in length, and 1 of the largest RNA viruses in terms of genome size. It has an envelope, with particles being round or elliptical, measuring 60 to 140 nm in diameter, primarily composed of RNA and proteins, and possesses 5 essential genes targeting the nuclear protein, viral envelope, matrix protein, and spike protein (S), as well as RNA-dependent RNA polymerase. The binding and entry of the virus are controlled by the key spike glycoprotein, with the S1 subunit containing 685 amino acids and the S2 subunit containing 588 amino acids. The S1 subunit possesses a receptor-binding domain (RBD) that binds to specific residues on the N-terminal helix of ACE2 (angiotensin-converting enzyme 2), allowing the virus to attach and facilitate entry into host cells, whereas the S2 subunit is responsible for membrane fusion. Studies have shown that there is a direct interaction between the host ACE2 receptor and viral spike protein^[1,2]^ hence, drugs or molecules that interact directly with ACE2 can prevent viral infection in cells. After the virus enters the host cells, the replication process requires the involvement of the main proteinase (3-chymotrypsin-like protease, 3CL, also known as the main proteinase) based on its irreplaceable role in the replication of SARS-CoV-2 and its relative conservation compared to the S protein, with a lower probability of natural mutations due to drug-induced mutations, making it a potential target for anti-SARS-CoV-2 therapy.^[3–6]^ COVID-19 patients mainly present with asymptomatic or mild symptoms (fever, cough, chest tightness, difficulty breathing, etc); however, patients with severe symptoms may experience severe respiratory infections, severe pneumonia, acute respiratory distress syndrome, multiple organ failure, and death.^[7]^ The COVID-19 pandemic has taken a huge toll around the world, and treatments are still being discovered or developed.

Since ACE2 receptors have been proven to be abundant in human lung epithelial cells, intestinal epithelial cells, olfactory nerve epithelial cells, nasal epithelial cells, and small intestinal epithelial cells, dietary intervention seems to be the most promising, as during normal food intake, these areas can all interact with and even intervene through diet. Long-term coffee consumption is beneficial to health. Coffee, which is an immunomodulator, is a major source of caffeine. Caffeine can prevent viral entry into cells, enhance phagocytosis by macrophages, improve breathing, stop cytokine storms, and inhibit the sequelae of multiorgan pathology.^[8]^ It is an inexpensive, safe, and widely used drug approved by the FDA, low-cost, and Drug safety. The immune-protective effects of caffeine can improve the treatment outcomes of patients with coronavirus infections. In the liver, caffeine can be metabolized by cytochrome *P*-450 enzyme and biotransformed into 3 active metabolites: inosine (81.5%), theobromine (10.8%), and theophylline (5.4%), which are then excreted through urine.^[9]^ At the same time, coffee contains various components related to the immune system, such as chlorogenic acid (CGA, the most abundant antioxidant in coffee, a type of phenolic acid), diterpenes (caffeine and coffee bean alcohols), and other substances that can inhibit oxidative damage. A diet rich in polyphenols and various antioxidants can elicit a positive human immune response^[10–12]^ and resist viral infections.^[13–15]^

Regular dietary behavior to prevent SARS-CoV-2 infection becomes an interesting issue. Particularly, coffee is 1 of the most commonly consumed beverages. And several studies have shown that coffee intake is associated with a reduced risk of COVID-19 and is associated with the severity of COVID-19.^[16–20]^ To our knowledge, there is currently no evidence-based medical evidence on whether intake coffee is beneficial for the prevention or treatment of COVID-19. Molecular docking technology is to predict the binding mode and affinity of small molecular ligands by studying their interactions with receptor biomacromolecules. Molecular docking methods play an important role in adverse reaction prediction, drug reuse, lead compound discovery, and new drug design.^[21,22]^ By virtual docking of potentially active substances with disease therapeutic targets, candidate compounds with specific interaction with target proteins can be found according to the docking results, so as to reveal the possible mechanism of action of drugs. Therefore, this study investigated the beneficial effects of frequent coffee consumption on the prevention or treatment of COVID-19 infection through a Meta-analysis, and provide relevant evidence-based medical evidence, and explored the mechanism of action of coffee components, such as caffeine and CGA, on the binding of SARS-CoV-2 3CL and human ACE2 by using molecular docking techniques. The results of this study may provide evidence for the public to prevent or treat COVID-19 infection by drinking coffee.

## 2. Materials and methods

This meta-analysis followed the methods of the Cochrane Handbook and complied with the 2009 preferred reporting items for systematic reviews and meta-analyses statement guidelines.^[23]^ We registered the review protocol with PROSPERO at the beginning (CRD 42024593482). No ethical approval or patient consent was required because all analyses were based on previous published studies.

### 2.1. Literature search and selection criteria

The search strategy involved using the subject terms “caffeine OR coffee” and “COVID-19 OR SARS-CoV-2,” combined with keywords to search databases such as PubMed, Cochrane Library, Embase, Web of Science, ClinicalTrials.gov, and CNKI, China Wanfang, China VIP, and Biomedical databases, with the search period from the establishment of the databases to August 1, 2024.

### 2.2. Data extraction

Data extraction was independently performed by 2 reviewers, including the following information: author, study location, publication year, number of participants, age and sex, and outcome measures. Disagreements were resolved by seeking approval from a third party.

### 2.3. Inclusion criteria

#### 2.3.1. Study types

We included cohort studies, randomized controlled trial (RCT), and cross-sectional studies and excluded nonclinical studies, reviews, animal experiments, and basic research.

#### 2.3.2. Outcome measures

Survival or death, olfactory function, number of infections (mild, moderate, and severe), hospitalization duration, antibiotic use during hospitalization, etc

### 2.4. Quality assessment

Quality assessment was conducted using the Cochrane Handbook for Systematic Reviews of Interventions, including the assessment of the risk of bias in the generation of random sequences, allocation concealment, blinding, data integrity, and selective reporting of study results. If all indicators were rated as low risk, the risk of bias was low; if 1 or more indicators were rated as unclear, the risk of bias was uncertain; and if 1 or more indicators were rated as high risk, the risk of bias was high.

### 2.5. Statistical methods

RevMan 5.4 software was used. Point estimates and 95% confidence intervals (CI) were calculated. Cochran *Q* test was used to assess heterogeneity among the studies, and the difference in heterogeneity was determined using the I^2^ value. When *I*^2^ ≤ 50%, a fixed-effects model was used when *I*^2^ ≤ 50 %; otherwise, a random-effects model was used; *P* < .05 was considered statistically significant.

### 2.6. Molecular docking

Crystal structures of the ACE2 receptor with ligand MLN-4760 (PDB: 1r4l) and 3CL receptor with ligand MI-23 (PDB: 7d3i) were downloaded from the Protein data bank (PDB). Molecular docking was performed using the glide module of Schrodinger 2018-1 software. Missing atoms and amino acid residues in the receptor were completed using the protein preparation module, followed by energy minimization. After removing heteroatoms and water molecules from the receptor and ligand, docking pockets were set as regions centered on the respective ligands with a radius of 10 Å. The 3-dimensional structures of CGA and caffeine in coffee were calculated using Lig Prep, and high-precision filtering was applied to screen for docking of these small molecules. Subsequently, dominant conformations were selected from numerous molecular conformations using docking score as the scoring function, and their 3-dimensional structures and molecular interactions with ACE2 and 3CL receptor proteins were analyzed. Because the docking score is the negative logarithm of the dissociation constant between the ligand and target protein, it reflects the affinity between the ligand and target protein. The greater the absolute value, the stronger is the affinity, suggesting a stronger inhibitory effect.

## 3. Results

### 3.1. Screening process

**Figure 1.**
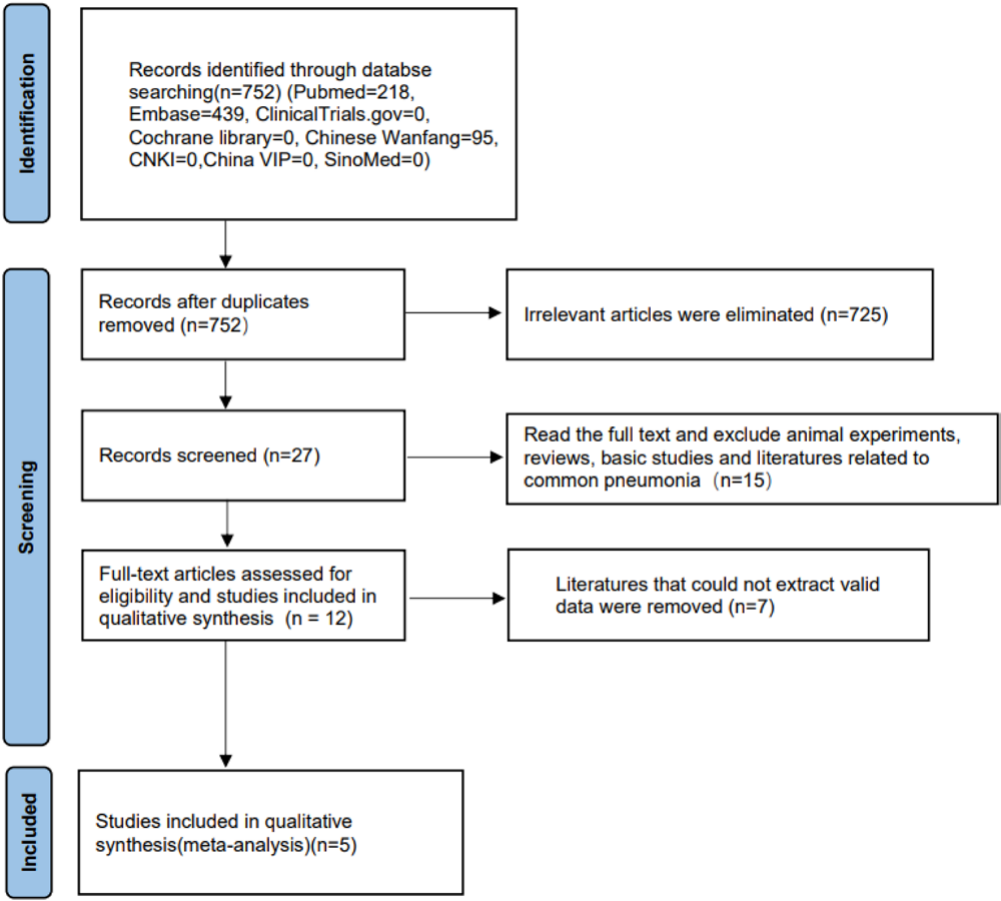
Flow diagram of study searching and selection process.

In the initial search, 752 articles were retrieved, and 725 articles that were completely unrelated to the topic were excluded after reviewing their abstracts. Further detailed reading of the full texts excluded 21 articles on animal experiments, reviews, and basic research. Finally, 5 studies with 39,290 participants were included in the meta-analysis based on data availability (Fig. 1).

### 3.2. Literature analysis and quality assessment

**Figure 2.**
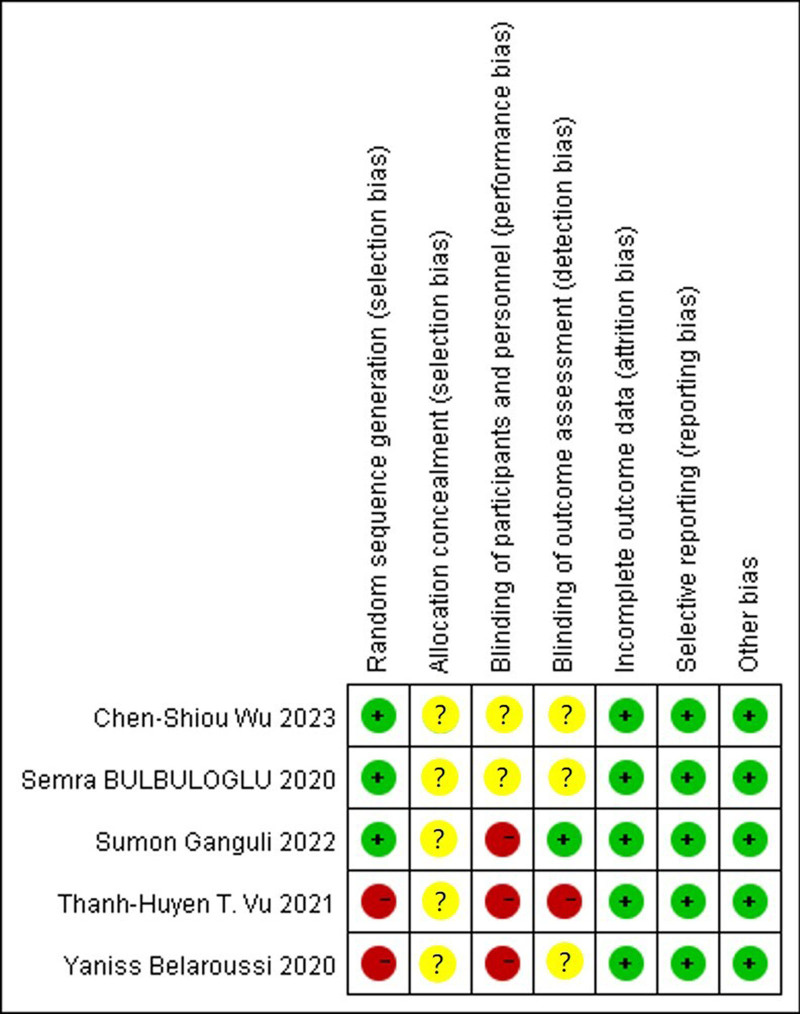
Risk of bias summary of included studies. Green, low risk of bias; yellow, unclear risk of bias; red, high risk of bias.

A total of 5 literature studies^[16–20]^ were included, with 37,900 participants in the experimental group and 1390 in the control group (Table 1). The included studies were assessed using the Cochrane risk assessment tool (Fig. 2).

**Table 1:** Basic characteristics of the Included studies.

Study (Author/yr)	Country	Number (male/female)	Average age	Enrollment criteria	Experimental group	Control group	Study type	Blinding	Outcome
Belaroussi/2020^[16]^	France	93 (47/46)	67.3	Patients with chest CT scan findings or nasopharyngeal swab-confirmed positive for COVID-19.	Drink a coffee containing 65 milligrams of caffeine every morning as symptomatic treatment for 2 weeks.	Corticosteroids and symptomatic treatment were administered every morning for 2 weeks.	NOT RCT	Not a randomized, double-blind study.	The primary endpoint is the clinical status of the patient on the 6th day after admission, including survival or death. The secondary endpoint includes hospital stay duration, secondary effects independent of the disease, and the use of antibiotics during hospitalization.
Bulbuloglu/2020^[17]^	Turkey	120 (58/62)	48.0	Nasopharyngeal swab confirmed as COVID-19 positive, with symptoms such as fever, cough, nasal congestion, sore throat, pneumonia, and headache, muscle weakness and other neurological symptoms, with moderate olfactory loss.	Sniff Turkish coffee for 1 hour, smelling for 1 to 2 minutes every 10 minutes, a total of 6 to 12 minutes of sniffing within the hour.	Perform clinical routine operations such as nasal irrigation with 0.9% isotonic saline for 1 hour, breathing exercises, and brushing teeth.	RCT	Randomized single-blind	The patient’s sense of smell recovery.
Wu/2023^[19]^	China	64 (34/30)	29.3	Healthy, non-COVID-19 infected volunteer serum.	Drinking coffee (including decaffeinated coffee).	Do not drink coffee.	RCT	Random	The seroprevalence of SARS-CoV-2 infection in the study participants.
Vu/2021^[18]^	American	37,988 (17,962/20,026)	57.4	Exposed UK biobank volunteers to COVID-19	From March 16, 2020, to November 30, 2020, on average, consumed at least 1 cup of coffee per day.	From March 16, 2020, to November 30, 2020, on average, consumed <1 cup of coffee per day.	Retrospective study	Non-randomized, non-blind	COVID-19 infection or not
Ganguli/2022^[20]^	Bangladesh	1025 (603/313,109 unclear)	35.9	Diagnosed as a positive case of COVID-19 by the COVID-19 testing laboratory through RT-PCR.	From November 2020 to March 2021, had a habit of drinking coffee.	From November 2020 to March 2021, had no habit of drinking coffee.	Retrospective study	Random	Whether hospitalized or not; combined with laboratory reports, divided into asymptomatic, mild, and severe patients.

COVID-19 = corona virus disease 2019, RCT = randomized controlled trial.

### 3.3. Main outcome indicators

#### 3.3.1. Overall efficacy

**Figure 3.**
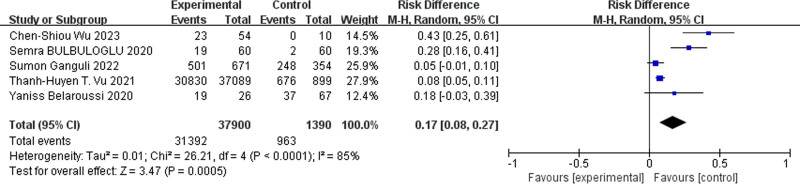
Forest plot for the meta-analysis of overall efficacy.

Five studies compared the prevention or treatment of COVID-19 between the daily consumption of more than 1 cup of coffee and no consumption or less than 1 cup of coffee. The combined analysis showed that regular coffee consumption significantly reduced the incidence of COVID-19 and improved the recovery rate from COVID-19 (RD = 0.17, 95% CI = 0.08–0.27, *P* < .0005, Fig. 3).

#### 3.3.2. Comparison of prevention effect

**Figure 4.**
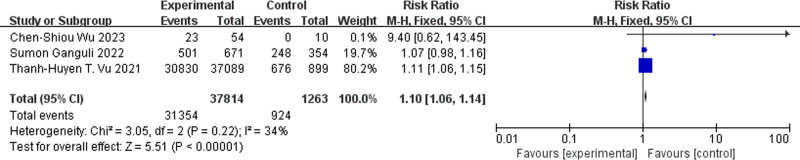
Forest plot for the meta-analysis of reducing COVID-19 infection rate. COVID-19 = corona virus disease 2019.

Three studies by Sumon Ganguli et al analyzed the association between specific dietary intake and the risk of COVID-19 infection and further examined the link between the consumption of diets containing coffee and the likelihood of infection. The combined analysis of the 3 studies indicated that the probability of COVID-19 infection among individuals who regularly consumed coffee was significantly lower than that among those who did not consume or consumed less coffee (RR = 1.10, 95% CI = 1.06–1.14, *P* < .00001, Fig. 4).

#### 3.3.3. Comparison of treatment effect

**Figure 5.**
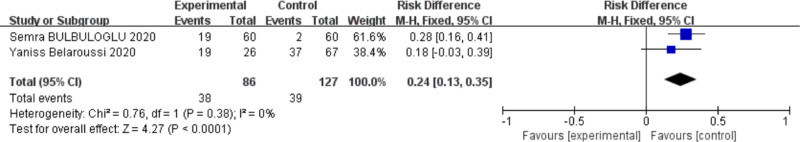
Forest plot for the meta-analysis of improving COVID-19 infection recovery rate. COVID-19 = corona virus disease 2019.

Two studies by Belaroussi et al were used to compare the physical recovery of COVID-19 patients who regularly consumed coffee. The results showed that the recovery rate of COVID-19 patients who frequently consumed coffee was significantly higher than that of those who did not consume or consumed less coffee (RD = 0.24, 95% CI = 0.13–0.35, *P* < .0001, Fig. 5).

### 3.4. Subgroup analysis

**Figure 6.**
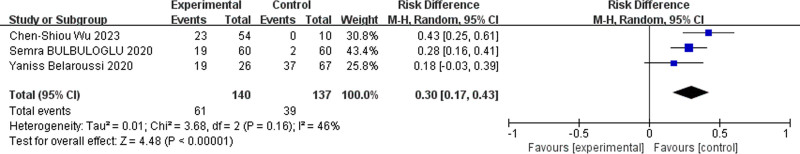
Forest plot for the meta-analysis of efficacy in experimental studies.

**Figure 7.**
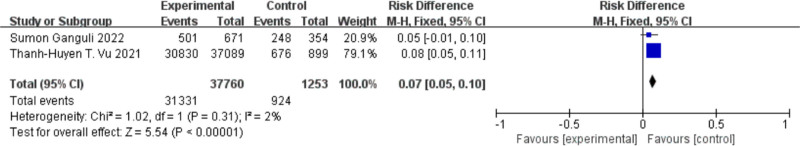
Forest plot for the meta-analysis of efficacy in cohort studies.

From the overall efficacy analysis, *I*^2^ = 85%, indicating significant heterogeneity in the overall efficacy. Further analysis based on the types of studies included suggested that heterogeneity could be attributed to differences in the types of studies included. Therefore, we conducted a subgroup analysis based on the different types of studies. Three experimental studies by Chen Shiou Wu et al (*I*^2^ = 46%, RD = 0.30, 95% CI = 0.17–0.43, *P* < .00001, Fig. 6) and 2 cohort studies by Sumon Ganguli et al (*I*^2^ = 2%, RD = 0.07, 95% CI = 0.05–0.10, *P* < .00001, Fig. 7) showed that regular coffee consumption significantly reduced the incidence of COVID-19 and improved treatment outcomes.

### 3.5. Molecular docking

**Figure 8.**
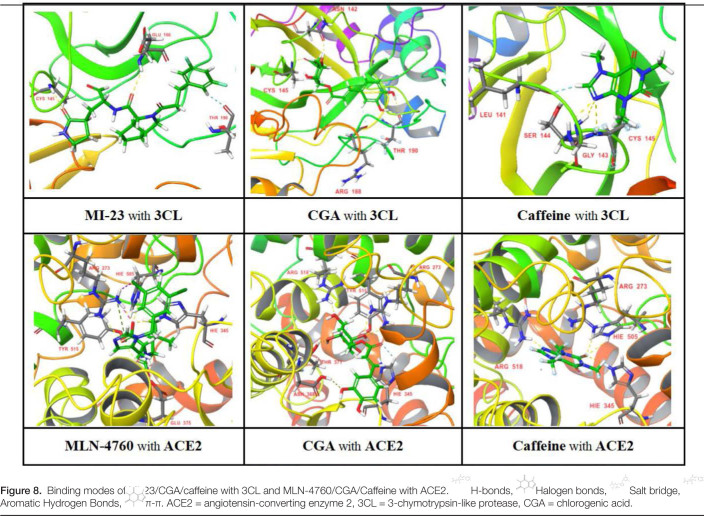


Coffee contains antioxidants such as caffeine, coffee bean alcohol, caffeine, coffee acid, and CGA, among which caffeine and CGA are present in higher amounts and may have a certain effect in preventing or treating COVID-19. Therefore, this study explored the possible mechanisms by which CGA and caffeine, the main components of coffee, prevent or treat COVID-19 through molecular docking. After docking, the binding of CGA and caffeine to the 3CL and ACE2 receptors is analyzed based on the scoring; to evaluate the accuracy and reliability of the results, molecular docking with known specific antagonists of the protein receptors is performed as a reference to further analyze possible mechanisms of action and binding patterns (hydrophobic interactions are not the focus of this study). The molecular docking results are shown in Tables 2 and 3 and the binding patterns of the receptors are shown in Figure 8.

**Table 2:**
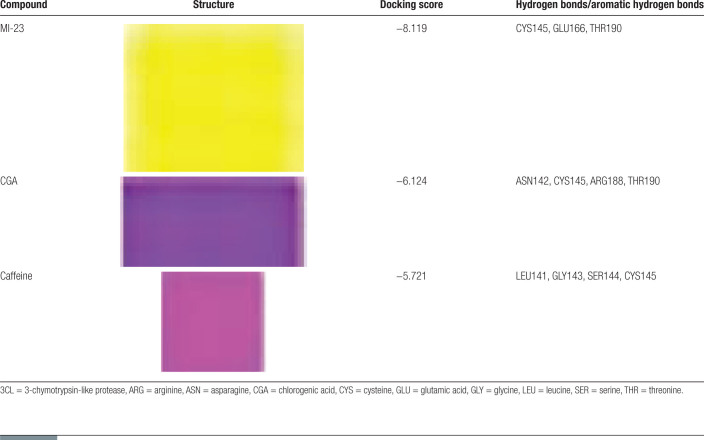
Docking of MI-23, CGA and caffeine with 3CL receptor.

**Table 3:**
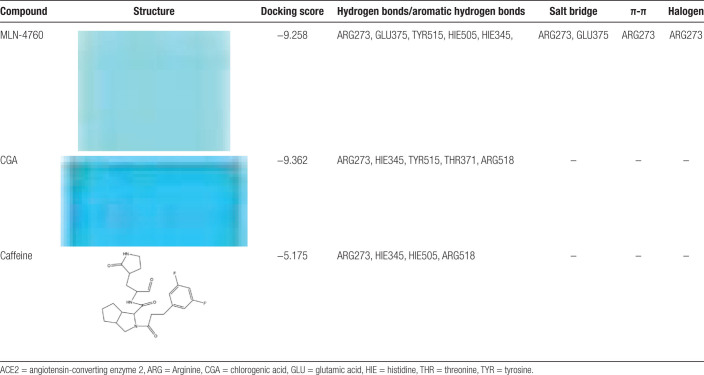
Docking of MLN-4760, CGA and caffeine with ACE2 receptor.

The potential mechanisms may involve strong hydrogen bonds between CGA and caffeine in coffee with Cys145 amino acid residues of the 3CL protein, which are key for inhibiting protease activity.^[7]^ This could prevent the production of nonstructural proteins that are necessary for viral replication and the formation of new virus particles, thereby blocking the formation of the RBD-ACE2 complex, reducing viral replication, and lowering the possibility of COVID-19 infection. Additionally, CGA and caffeine in coffee can form strong hydrogen bonds with amino acid residues such as ARG273 and HIE345 in the ACE2 receptor protein, with CGA showing a better docking score (−9.362) than the specific inhibitor MLN-4760 (−9.258) and a stronger binding effect. This could potentially compete with the virus to antagonize ACE2 binding sites, thereby inhibiting the entry of SARS-CoV-2 into host cells^[24]^ and is beneficial for the recovery of COVID-19 patients. The results of molecular docking showed that compared with the specific inhibitor MI-23 and MLN-4760, caffeine and CGA, 2 bioactive components with higher content in coffee, both had the ability to inhibit the entry/replication of SARS-CoV-2. Compared to caffeine, CGA has a better ability to block SARS-CoV-2 infection/replication.

## 4. Discussion

This meta-analysis included 5 studies with 39,290 participants to compare the beneficial effects of regular coffee consumption against less frequent or nonconsumption in preventing or treating COVID-19, and to explore possible mechanisms. A meta-analysis showed that regular coffee consumption was associated with a lower probability of positive COVID-19 results and higher recovery rate among positive patients. Molecular docking showed that CGA and caffeine present in coffee could combine with key amino acid residues of ACE2 or 3CL proteins to form hydrogen bonds. Studies have reported that countries with high consumption of foods with strong antioxidant or ACE inhibitory activities, such as raw or fermented cabbage, have lower COVID-19 mortality rates.^[25]^ Therefore, CGA and caffeine may be the key chemical substances present in coffee, potentially inhibiting the activities of 3CL and ACE2 and having beneficial effects in reducing the risk of COVID-19.

To date, most countries have lifted strict lockdown restrictions and have coexisted with COVID-19. Therefore, preventive dietary behaviors against COVID-19 have become a concern in daily life. Coffee is 1 of the most widely consumed beverages in the world, and people currently drink it mainly because caffeine can improve athletic performance, reduce fatigue, and enhance alertness and consciousness. However, caffeine has also been proven to be an effective anti-inflammatory and immune modulator, with antioxidant or repair mechanisms and anti-inflammatory and anticancer capabilities^[26–28]^。Yamazaki and Tagaya^[29]^ reported the antiviral activity of caffeine against poliovirus, influenza virus, HSV-1, and cowpox viruses. Olson and Consigli^[30]^ reported that caffeine inhibits the replication of Newcastle disease virus by suppressing the synthesis of viral RNA and proteins in infected cells. Caffeine and other methylxanthines have also been shown to inhibit the replication of infectious HIV-1 strains, with the integration stage of the HIV-1 life cycle as a target for caffeine.^[31]^ Coffee is not only a major source of caffeine but is also rich in dozens of other components related to the immune system^[32]^ and is a major source of total polyphenol intake, including CGA, cafestol, and other antioxidant compounds such as melanin and trigonelline.^[33–37]^ CGA polyphenols can interfere with human blood pressure, lipids, blood sugar, and insulin resistance, effectively improving metabolic syndrome, and have antiviral activity, such as inhibition of the integrase of RSV, hepatitis B virus, and HIV.^[38–40]^ Furthermore, coffee intake is positively correlated with inflammatory biomarkers such as C-reactive protein, interleukin-6, and TNF-α,^[41–48]^ which are also related to the severity and mortality of COVID-19.^[49]^ A large prospective cohort study in the United States showed a negative correlation between coffee intake and causes of death from chronic respiratory diseases, pneumonia, and influenza.^[50]^ Other cohort studies have also reported a negative correlation between coffee intake and the risk of death from respiratory diseases (pneumonia, influenza, chronic obstructive pulmonary disease, and related symptoms).^[51,52]^ A Japanese study also showed that coffee intake was associated with a lower risk of pneumonia in the elderly.^[53]^ These findings suggest that coffee may have a preventive effect on chronic and acute respiratory diseases, which is consistent with the conclusions of this study.

The results of this study suggest that coffee may have an impact on COVID-19 and do not guarantee that drinking coffee will prevent infection with SARS-CoV-2. The main reasons for this are as follows: Currently, there are relatively few high-quality RCT trials on the prevention and treatment of COVID-19 using coffee. The present study included 5 articles, which is a small number and there is a large heterogeneity; therefore, publication bias was not analyzed and may exist to some extent. Coffee intake in the studies was measured in cups without specifying the exact amount, and it is difficult to determine the content of caffeine, CGA, and other antioxidants in each cup of coffee. The type of coffee, origin, and brewing method also affected the results. Owing to confounding variables and inherent limitations, the observational cohort studies included in this study failed to establish a potential causal relationship between a coffee-containing diet and COVID-19. For example, when studying the relationship between lifestyle and COVID-19, socioeconomic status, national geography, and cultural level may affect lifestyle and health status, but related studies did not consider them as potential confounding factors. Molecular docking results indicated that CGA and caffeine in coffee can form hydrogen bonds with ACE2 or 3CL proteins, which have high affinity, but have not been verified for their binding conformation stability with receptor proteins through molecular dynamics simulations, nor have they been validated at the cellular and molecular levels. There are more than 1000 components in coffee, most of which have not been identified. Therefore, the effects of other chemicals in coffee on COVID-19 still need to be further explored. Therefore, future clinical trials require larger sample sizes and more high-quality multicenter RCT, as well as more in vitro and in vivo studies, to confirm the clinical efficacy and mechanisms of action. The most effective methods for preventing or treating COVID-19 are national health department recommendations, consultation with a professional doctor, or seeking medical treatment when experiencing discomfort.

## 5. Conclusion

In summary, we explored the possible beneficial effects of coffee consumption on COVID-19 through meta-analysis, and the mechanism of action may be that CGA/caffeine in coffee may be related to the formation of hydrogen bonds by key amino acid residues such as ARG273/ HIE345 of ACE2 and CYS145 of 3CL by molecular docking. In particular, coffee is 1 of the most commonly consumed beverages and a common dietary habit. It is therefore recommended that drinking coffee may be adopted in the new COVID-19 protection guidelines to limit the risk of infecting SARS-CoV-2.

## Author contributions

**Conceptualization:** Yun-Li Duan.

**Formal analysis:** Yun-Li Duan, Yu Wang.

**Investigation:** Yong-Zheng Fan.

**Project administration:** Yu Wang.

**Writing – original draft:** Yong-Zheng Fan, Yun-Li Duan, An-Na Zhang.

**Writing – review & editing:** Yong-Zheng Fan, Yu Wang.
